# Effect of milk exposure on the redox profile of *Caenorhabditis elegans*

**DOI:** 10.1038/s41598-024-52254-6

**Published:** 2024-01-22

**Authors:** Ingrid Laíse Silvestre de Oliveira, Giovanna Melo Martins Silva, Cesar Orlando Muñoz Cadavid, Danielle Cavalcanti Sales, Katya Anaya, Riva de Paula Oliveira, Adriano Henrique do Nascimento Rangel

**Affiliations:** 1 Escola Agrícola de Jundiaí, Unidade Acadêmica Especializada em Ciências Agrárias, Universidade Federal do Rio Grande do Norte, Macaíba, RN 59280-000 Brazil; 2 Rede Nordeste de Biotecnologia (RENORBIO), Universidade Federal do Rio Grande do Norte, Natal, RN 59078-970 Brazil; 3 Faculdade de Ciências da Saúde Do Trairí (FACISA), Universidade Federal do Rio Grande do Norte, Santa Cruz, , RN Brazil; 4 Departamento de Biologia Celular E Genética, Universidade Federal do Rio Grande do Norte, Natal, RN 59078-970 Brazil

**Keywords:** Biological techniques, Animal disease models

## Abstract

The consumption of bovine milk and its derivatives is associated with inflammation, gastrointestinal disorders and the development of diseases in humans. Most studies related to milk effects are based on either clinal trials or experimental models such as mice and cell cultures. In this study we present the nematode *Caenorhabditis elegans* as an alternative model to evaluate the effects of milk on oxidative stress in other animal models. The toxicological effect of 20% milk exposure for 8 h on *C. elegans* was evaluated by progeny quantification, body size and pharyngeal pumping rate. Treating the worms with milk did not affect the worms brood size but interfered with their fecundity by delaying the average number of eggs in the first day of oviposition when compared to the control group. The size of worms treated with milk were significantly smaller compared to control. The pharyngeal pumping rate of milk-treated animals was not significantly different compared to untreated animals. Taking together, the results suggest that 20% milk treatment is not toxic for the worms but induces a minor delay achieving its adulthood and therefore its reproduction period. Milk exposure did not reduce the worms’ survival under stress conditions and increase endogenous ROS levels. This study contributes to characterize the effects of milk exposure on the *C. elegans* nematode.

## Introduction

Milk is considered a vital part of a healthy and balanced diet because of its nutrition value. Bovine milk and its derivatives have been the target of criticism, precisely because their consumption is associated with biomarkers of inflammation, gastrointestinal disorders and disease development^1,2^. On the other hand, studies have demonstrated that dairy intake produces significant and substantial suppression of oxidative and inflammatory stress^1,3,4^, and milk proteins are capable of attenuating the reactivity of reactive oxygen species (ROS) and increasing the antioxidant enzyme activities^4,5^. The administration of milk with high CLA (conjugated linoleic acid) to rats resulted in beneficial effects on lipid metabolism, inflammation and oxidative stress in the liver^6^.

Most studies related to milk effects are based on either clinical trials or experimental models such as mice and cell cultures. Here we present *Caenorhabditis elegans* as an alternative model to evaluate the effects of milk on oxidative stress in other animal models. *C. elegans* is an excellent model organism for experimental research involving metabolic disorders, as 60-80% of human genes have an ortholog in the *C. elegans* genome and 40% of known genes and associated with human diseases have orthologs in *C. elegans*^7,8^. In addition, due to the difficulty of using metabolic processes in humans in real time, studies are based on animal models which can contribute to clarifying the molecular pathways involved in human diseases at the metabolic or genomic level in vivo. As *C. elegans* has signaling pathways that are highly conserved to study oxidative stress, we sought to assess whether *C. elegans* could be used as a model to study the interference of milk in the levels of reactive oxygen species and in the resistance capacity against stress conditions. Therefore, the objective herein was to evaluate the effect of exposure to milk on the levels of reactive oxygen species and on the stress resistance of *Caenorhabditis elegans*.

## Methods

### Milk samples

**Figure 1:**
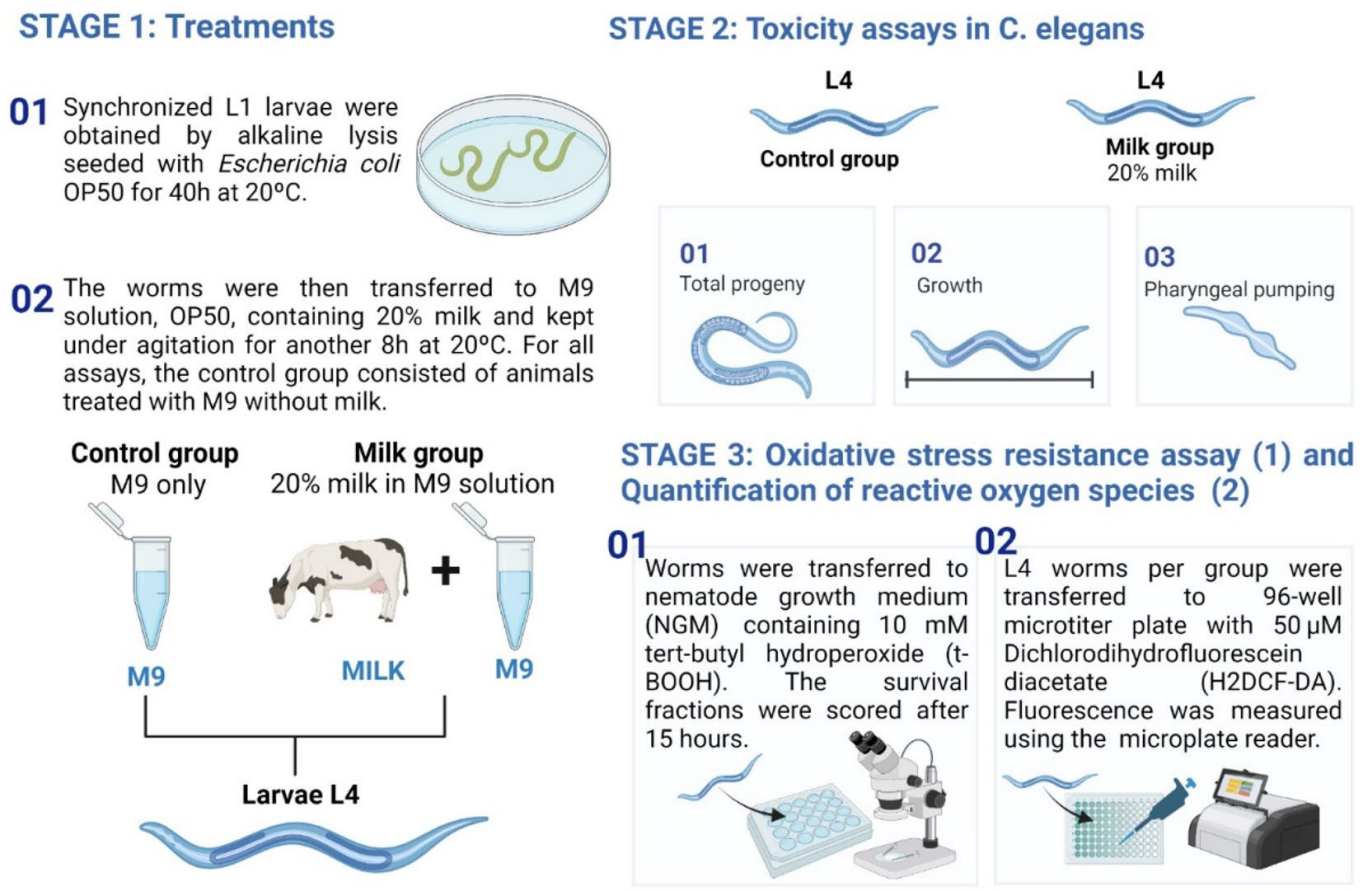
Preparation of treatments and tests of toxicity, oxidative stress resistance and quantification of reactive oxygen species.

Figure 1 illustrates the methodology of the study with *C. elegans*. Four samples of pasteurized cow milk from different herds were used (Table 1). The samples were stored in small aliquots at − 20 °C until used for experiments. This study was approved by the Animal Research Ethics Committee of the Federal University of Rio Grande do Norte, under protocol No. 022/2020.

**Table 1:** Composition of pasteurized bovine milk samples used in the experiment, obtained from 4 different herds.

Sample	Milk composition (%)				
	Fat	Protein	Lactose	Solids-not-fat	Total solids
#1	1.65	2.95	4.42	8.04	9.69
#2	3.65	3.08	4.63	8.43	12.08
#3	3.52	2.61	3.92	7.14	10.66
#4	2.60	3.11	4.66	8.48	11.08

### *Caenorhabditis elegans* maintenance and treatment

*Caenorhabditis elegans* strain used in this work was N2 wildtype for all tests. Synchronized L1 larvae were obtained by alkaline lysis (10 M NaOH, 2% sodium hypochlorite) and grown on Nematode Growth Medium (NGM) plates (0.25% Tryptone, 0.3% NaCl, 1.5% Agar, 1 mM CaCl, 1 mM MgSO_4_, 25 Mm KPO_4_ pH 6.0, and 5 µg/mL Cholesterol) seeded with *Escherichia coli* OP50 for 40 h at 20 °C unil L3 stage^9^. The worms were then transferred to M9 solution (20 mM M KH_2_PO_4_, 40 mM Na_2_HPO_4_, 850 mM NaC and 1 mM MgSO_4_) containing 20% milk and *E. coli* OP50 for another 8 h at 20 °C under agitation until L4 stage. For all assays, the control group consisted of animals treated with M9 + *E. coli* OP50 without milk.

### Toxicity assays in *C. elegans*

The toxicity to milk exposure was assessed by measuring brood size, growth and pharyngeal pumping. To determine total progeny, ten L4 worms, previously treated with 20% milk or M9 solution were placed on individual NGM plates. During the egg-laying period, nematodes were transferred onto new plates every 24 h for five days until the end of the reproductive period. The F1 progeny from each individual worm was counted. The total progeny numbers for each plate were calculated and divided by the number of animals. For growth, 30 worms from each group after treatment were photographed using the AmScope MU300 Digital Camera connected to an optical microscope (Axio Imager Z2, Zeiss, NY, USA). Body area and length was determined by using the ImageJ software. The average pharyngeal pumping rate was calculated by three 20-second intervals of ten worms per group. All experiments were conducted in triplicates.

### Oxidative stress resistance assay

After milk treatment, worms were transferred to solid NGM containing 10 mM tert-butyl hydroperoxide (t-BOOH). This medium was poured into a 24-well plate. Each well was then seeded with *E. coli* OP50. For each condition, which included the control and the 20% milk condition, we utilized five wells, each containing ten nematodes. The survival fractions were scored after 15 hours. The experiment was carried out in three independent experiments.

### Quantification of reactive oxygen species (ROS)

ROS levels were measured using H2DCF-DA (2′,7′-Dichlorodihydrofluorescein diacetate) as described previously^10^. After milk treatment, approximately forty L4 worms per group were transferred to PBS+ 1% Tween-20 on a 96-well microtiter plate, to which 50 μM H2DCF-DA was added. Measurements were performed in triplicate in a multilabel microplate reader GloMax®-Multi Detection System (Promega, Wisconsin, USA), with excitation at 490 nm and emission at 510–570 nm, and the mean values were calculated. Readings were performed every 30 min for 2 h.

### Statistical analysis

Data analysis was performed using descriptive statistics using mean and standard deviation. All data obtained were submitted to the statistical program GraphPad Prism® version 6.01 (USA) for graphing and data analysis. Non-parametric data were analyzed by ANOVA to compare groups. The Student’s t-test was used for data with normal distribution. Significance was considered at 5%.

## Results and discussion

### Evaluation of toxicological effects of milk in *C. elegans*

Milk supplementation has been already employed in *C. elegans* as an alternative strategy to develop an axenic growth medium named *C. elegans* Habituation and Reproduction (CeHR) medium^11^. Interestingly, this protocol uses 20% of milk in the final volume of a liquid medium. Here, we also exposed *C. elegans* to 20% milk but differently from the procedures previously described for the axenic CeHR medium, we first grew L1 worms on regular solid NGM medium for 40 h and then submitted them at L3–L4 transition state for 8 h in order to evaluate the effect of exposure to milk on the levels of reactive oxygen species and on the stress resistance.

**Figure 2:**
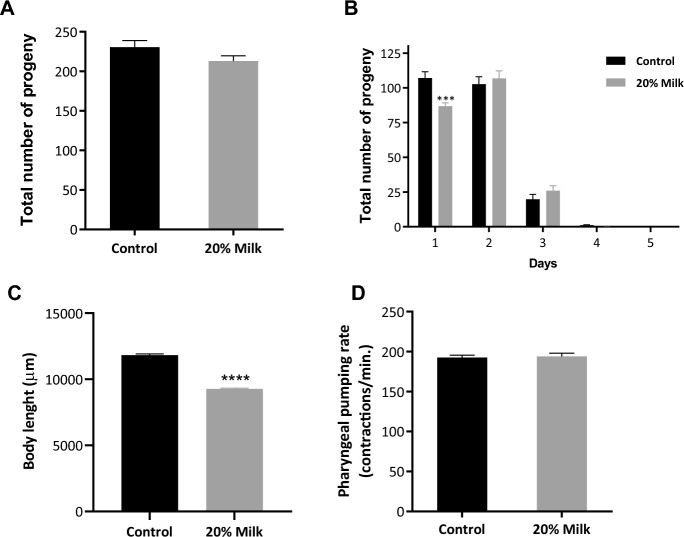
Effect of 20% milk treatment on *C. elegans* toxicological parameters. L3 wild-type animals were treated with 20% milk for 8 h until they reached L4 stage and then toxicity was assessed by measuring total brood size (**A**), number of embryos laid per day (**B**), body size (**C**) and pharyngeal pumping (**D**). (**A**) In terms of total number of eggs laid, there is no statistical difference between control and treated worms. (**B**) On the first day of oviposition, the number of eggs laid by the animals treated with milk was significantly reduced. ***p = 0.0006 by 2-Way-ANOVA. (**C**) Worms treated with 20% have body length significantly reduced. ****p < 0.0001 by Student’s t-test. (**D**) There is no statistical difference in pharyngeal pumping rate between control and treated worms.

Since changing the worms from agar to liquid axenic media is associated with phenotypical alterations such as delayed development, and reduced fecundity^12,13^, we first evaluated the effect of milk ingestion on these two biological parameters. Treating the worms with 20% milk for 8 hours did not affect the worm's brood size (Fig. 2A). However, milk exposures interfered with their fecundity by delaying the average number of eggs on the first day of oviposition when compared to the control group (Fig. 2B). Using an approach based on bacterial ring assay to evaluate the effect of chemical exposure on *C. elegans* chemotactic behavior and reproduction over the three-day life cycle, Worku and Gerald^11^ observed that both colostrum and non-fat milk increased migration and reproduction. Interestingly, we also observed that the worms treated with milk were significantly smaller compared to control (Fig. 2C). These results also suggest that even a short period exposure of 8 hours incubation in milk-supplemented M9 is able to influence the worms’ development.

We also measured the pharyngeal pumping rate in L4 animals in order to verify whether milk treatment alters feeding behavior. The pharyngeal pumping rate of milk-treated animals was not significantly different compared to untreated animals (Fig. 2D). Taking together, the results suggest that 20% milk treatment is not toxic for the worms but induces a minor delay achieving its adulthood and therefore its reproduction period.

### Effect of milk treatment on oxidative stress resistance in *C. elegans*

Several studies have already highlighted the antioxidant potential and response to oxidative stress of milk and dairy protein intake^1,3–5,14^. However, few studies have explored these effects in the *C. elegans* animal model. Here, we evaluated how 20% milk treatment would affect *C. elegans* stress resistance.

**Figure 3:**
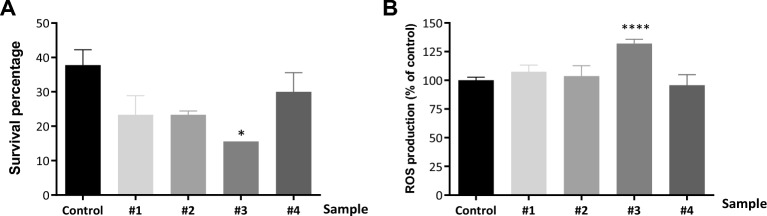
Effect of 20% milk on ROS production and oxidative stress resistance of *C. elegans*. Stress resistance assay. L3 wild-type animals were treated with 20% milk for 8 h until they reached L4 stage and transferred to NGM supplemented with 10 mM t-BOOH to induce oxidative stress conditions. Animal survival was scored after 24 h. Assays were performed in triplicate and expressed as mean ± SEM. *(*P* < 0.05) Student’s t-test (**A**). ROS quantification. L3 wild-type animals were treated with 20% milk for 8 h and the production of ROS was measured using the H2DCF-DA probe. Assays were performed in triplicate and expressed as mean ± SEM. ***p < 0.001 by Student’s t-test (**B**). #1, #2, #3 and #4: milk samples with different compositions.

The composition of bovine milk can vary according to several factors, such as genetics, age and physical condition, but it is mainly influenced by the animals' diet. Since milk composition can vary, we tested different samples (Table 1). Although the average survival fraction of milk-treated worms was reduced compared to control group, statistical analysis showed that there was no significant difference, expect for milk sample #3 (Fig. 3A).

**Figure 4:**
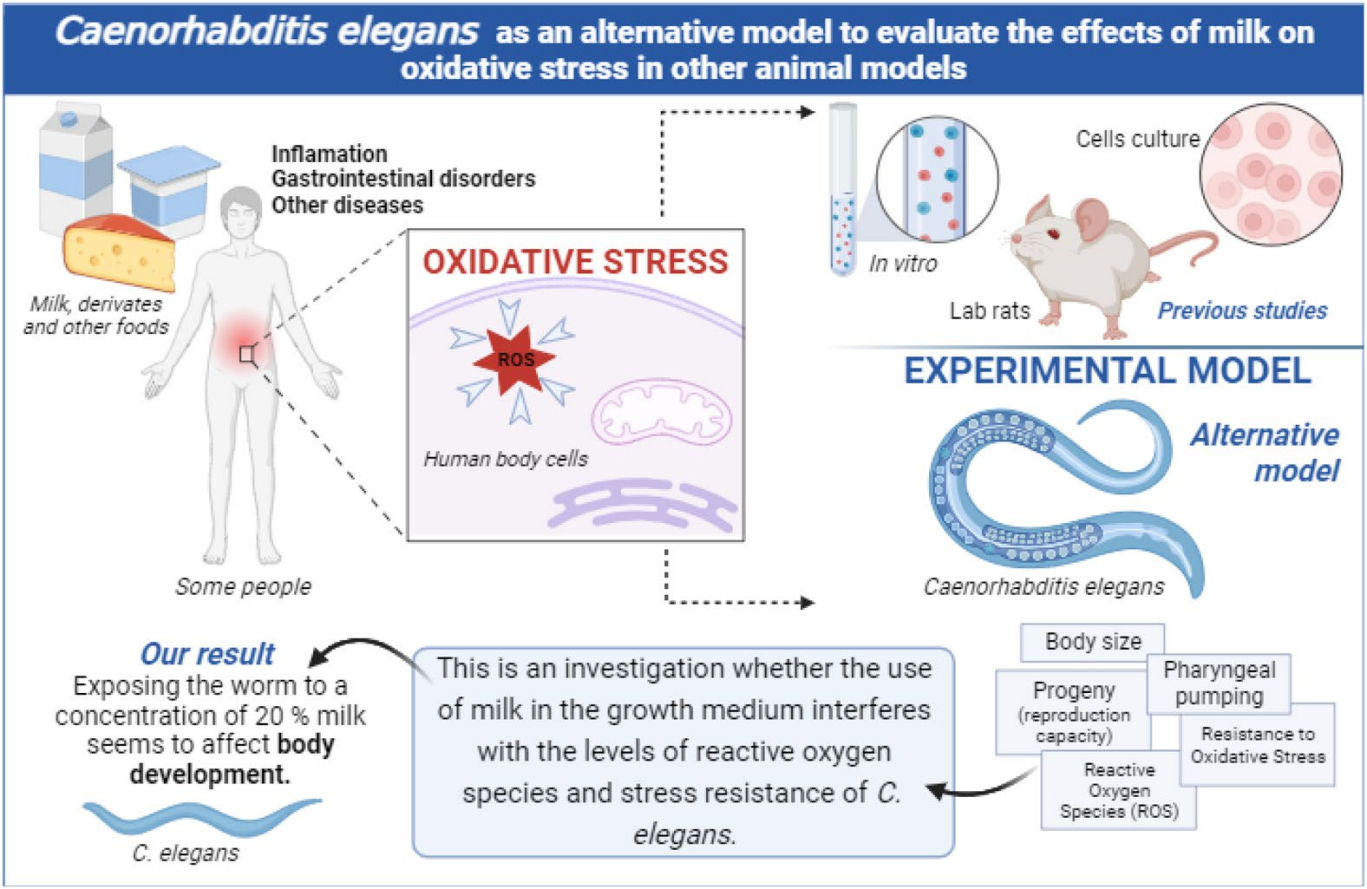
Illustration of the study, variables and main results findings.

In order to associate these results with a possible oxidant effect induced by milk treatment, we measured worms’ endogenous ROS production. We observed that ROS production in milk-treated worms was not different when compared to the control group except for sample #3 (Fig. 3B). Interestingly, only worms treated with this sample showed significantly increased ROS levels, which is the same milk sample that causes reduced oxidative stress survival. Xing et al. observed that worms treated with lactose showed reduced lifespan, which was completely rescued by the co-administration of the antioxidant N-Acetyl-L-cysteine (NAC), indicating that redox stress could be, at least in part, responsible for the lactose-induced senescence^15^. Interestingly, the percentage of lactose in sample #3 is the lowest compared to other samples used in our work, which contradicts what was observed by Xing et al.^15^. Protein fraction present in milk is also able to interfere in ROS production as shown by an ex vivo study carried out by Albenzio^16^. The study demonstrates that lower ROS levels were found in cells incubated with α-lactalbumin and β-lactoglobulin, while the highest level was found after incubation with β-casein isolated from bovine milk when compared to goat and sheep species. Thus, the protein composition of the studied milk may influence the individual’s response to oxidative stress. When using the in vivo *C. elegans* model from the tested samples, the milk treatment generally does not affect resistance to oxidative stress and does not increase ROS levels under oxidative stress conditions. The main results of the study are illustrated in Fig. 4.

Although most of the studies cited in the literature claim reduced oxidative stress by ingestion of dairy products, the present study was not able to find a reduction in ROS for the evaluated samples. It is important to emphasize that as there are few works using milk in *C. elegans*, the comparisons which can be made are based on studies with other experimental models, which despite being widely used, have different metabolic responses.

## Conclusion

Under the conditions of this study, exposure to 20% milk does not seem to decrease resistance under stress conditions and does not interfere with endogenous ROS levels in wild-type *C. elegans*.

## Data Availability

Data will be made available upon request to the corresponding author.
